# An RNA-Directed Gene Editing Strategy for Attenuating the Infectious Potential of Feline Immunodeficiency Virus-Infected Cells: A Proof of Concept

**DOI:** 10.3390/v12050511

**Published:** 2020-05-05

**Authors:** Brian G. Murphy, Tatiana Wolf, Helena Vogel, Diego Castillo, Kevin Woolard

**Affiliations:** 1 Department of Pathology, Microbiology & Immunology, School of Veterinary Medicine, University of California Davis, Davis, CA 95616, USA; castillodiego@yahoo.com (D.C.); kdwoolard@ucdavis.edu (K.W.); 2 School of Veterinary Medicine, University of California Davis, Davis, CA 95616, USA; twolf@ucdavis.edu (T.W.); hvogel@ucdavis.edu (H.V.)

**Keywords:** feline immunodeficiency virus, FIV, eradication, gene editing, CRISPR Cas9, latency, reservoir

## Abstract

Modern antiretroviral therapy for immunodeficiency viruses, although remarkably effective in controlling viral transcription, and overt virus-associated morbidity, has failed to absolutely eradicate retroviruses from their infected hosts as a result of proviral integration in long-lived reservoir cells. Immunodeficiency virus-infected patients are therefore consigned to lifelong antiviral therapy as a means to control viremia, viral transmission, and infection-associated morbidity. Unfortunately, lifelong antiviral therapies can be difficult for patients to continuously maintain and may be associated with therapy-specific morbidities. Patient advocates have argued for new methods to achieve retroviral eradication. As a proof-of-concept study, a lentivirus-delivered RNA-directed gene editing strategy was utilized in a series of in vitro experiments in an attempt to attenuate the feline immunodeficiency virus (FIV) proviral load, viral transcription, and production of infectious virions. We found that a feline T lymphocyte cell line (MCH5-4) treated with an FIV-specific clustered regularly interspersed short palindromic repeats (CRISPR)-associated protein 9 (Cas9) gene editing tool resulted in a reduction of cell-free viral RNA relative to control cells. Decreased infectious potential was demonstrated in a two-step FIV infection study—naïve MCH5-4 cells infected with cell-free FIV harvested from FIV-infected and CRISPR lentivirus-treated cells had less integrated proviral DNA than control cells. This study represents the initial steps towards the development of an effective method of proviral eradication in an immunodeficiency virus-infected host.

## 1. Introduction 

The advent of combinatorial antiretroviral therapy (ART) almost 25 years ago has been remarkably effective in attenuating the viremia, viral transmissibility, and morbidity associated with immunodeficiency virus infections [1]. Nevertheless, lifelong retroviral infection is the likely outcome due to efficient and stable integration of a DNA copy of the viral genome (provirus) into long-lived reservoir cells early on during infection [2,3,4]. Cells infected with a transcriptionally silent and integrated (latent) provirus typically fail to “advertise” their infected status and therefore remain undetected by the host’s adaptive immune response. Integrated proviral genomes are efficiently copied and passed on to daughter cells, ensuring the continued presence of a persistent population of virus-infected cells within the host.

For human patients infected with the human immunodeficiency virus (HIV), permanent integration of HIV into the host genome results in a continued risk of viral reactivation, even following ART [5]. In addition, patients on long-term ART often have comorbidities including cardiac, renal, bone, and neurologic disorders [6]. Comorbidities may be the result of the ART itself or persistent, low level expression of viral proteins. In most cases, the abrupt cessation of ART typically results in a reemergence of viremia followed by immunodeficiency disease progression. Therefore, the persistent population of latently infected cells, distributed in specific anatomic locations throughout the host, forever precludes the cessation of ART. In order to avoid a reemergent viremia, HIV-infected patients have been relegated to lifelong ART therapy. 

A variety of experimental strategies have been explored in an attempt to eradicate this population of latently infected cells. A medical strategy referred to colloquially as “shock and kill”, involves therapeutic reactivation of the integrated viral promoter (antilatency therapy—the “shock”) resulting in activation of viral gene expression [7]. Viral antigens subsequently deposited on the infected cells’ surface membrane are purported to facilitate immune recognition and removal of the virus-infected cell (the “kill”). Unfortunately, therapeutic reactivation of a significant proportion of the latently infected cells within the host has been difficult to achieve in vivo [8]. Another strategy that has been attempted is the modification of the host’s chemokine receptor repertoire to eliminate the viral co-receptors, like CCR5 or CXCR4, from virus-susceptible leukocytes [9]. A variety of methods have been proposed to accomplish this including bone marrow transplantation with CCR5 restricted cells [10], or gene edited modification of the host cells. 

Several different engineered nuclease systems have been utilized to disrupt, or edit, target genes including zinc finger nucleases, transcription activator-like effector nuclease (TALEN), and clustered regularly interspersed short palindromic repeats (CRISPR)-associated protein 9 (CRISPR Cas9) [9,11,12,13]. CRISPR technology has the advantage of precise sequence-specific targeting of the nuclease enzyme. The CRISPR Cas9 system evolved naturally in bacteria as a prokaryotic weapon against invading pathogens and has been recently repurposed by scientists into an efficient and specific method of genome editing [14]. Target-specific sequences, so-called guide RNA (gRNA), precisely localize the Cas9 nuclease, while the actual double-stranded cleavage site is demarcated by adjacent conserved sequences called proto-spacer adjacent motifs (PAMS) [15]. The nuclease-induced double stranded breaks are subsequently repaired by standard cellular repair mechanisms—either homologous or non-homologous recombination [15]. For HIV-infected cells, excision of the coding sequence of the integrated lentiviral genome from the host cell genome has been successfully demonstrated using a CRISPR technique targeting the conserved 5′ and 3′ long terminal repeats (LTRs) of HIV [16,17]. Importantly, gene editing-mediated proviral excision resulted in neither genotoxicity nor off target editing in latently infected microglia and T cells [5]. However, more recent studies using CRISPR-mediated genetic modification in an HIV infection model have identified two opposite outcomes- inactivation of HIV-1 and acceleration of viral escape, potentially limiting the use of the CRISPR Cas9 gene editing strategy for lentiviral infections [18].

Feline immunodeficiency virus (FIV) infection of cats is the only naturally occurring model of lentivirus-mediated immunodeficiency disease [19]. FIV shares genomic organization, receptor usage, lymphocyte tropism, induction of immunodeficiency, and increased susceptibility to cancer with HIV [20]. As is true for HIV, FIV causes a progressive depletion of CD4+ T cells and collapse of the host’s adaptive immune system, eventually resulting in a terminal AIDS-like syndrome referred to as feline AIDS, or FAIDS [21,22]. Here we explored a method using the gene editing technology CRISPR to reduce the proviral load, cell-free viral RNA and infectious virion production of a population of FIV-infected feline CD4+ T cells. We hypothesized that in an in vitro culture system, an FIV-targeting CRISPR Cas9 editing tool would excise the FIV coding region resulting in a reduction of integrated provirus, cell-free virus, and viral infectivity. 

## 2. Materials and Methods 

### 2.1. Gene Editing Strategy 

**Figure 1:**
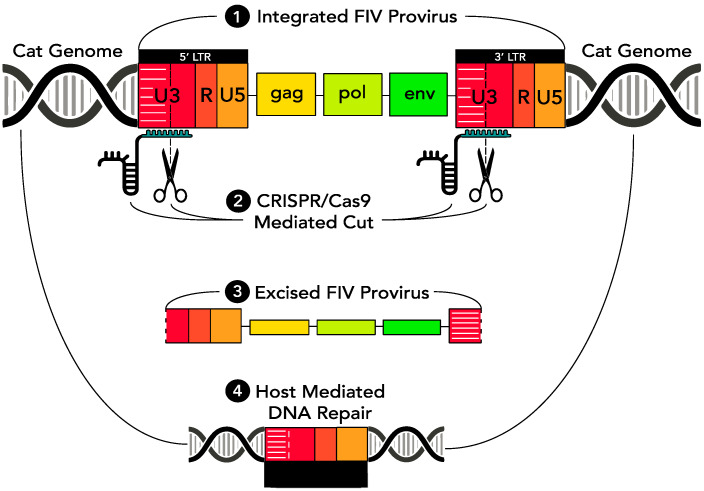
Clustered regularly interspersed short palindromic repeats (CRISPR)-associated protein 9 (Cas9) gene editing strategy.

As a means of eradicating replication competent provirus from virally infected cells, a CRISPR Cas9-based gene editing strategy was introduced through a series of in vitro experiments into FIV-infected cells via a replication defective lentivirus vector. The CRISPR Cas9 nuclease was engineered to concurrently target two sites within the FIV proviral U3 region, both of which are encoded within the 5′ and 3′ LTR (which are direct repeats). Based upon in vitro studies using HIV [5], we predicted that sequence-specific cleavage within both the 5′ and 3′ LTRs would result in release of the entire FIV coding region (*gag-env*) from the infected host cell genome. Subsequent host cell-mediated DNA repair mechanisms would then repair the Cas9 nuclease-induced double stranded cuts by aligning and annealing the incised 5′ and 3′ LTRs. In time, the excised, approximately 9000 bp proviral episome would likely degrade, resulting in a loss of viral replication competence and leaving a remnant functionally disabled single FIV LTR integrated within the host cell genome (Figure 1).

### 2.2. Lentiviral Vectors 

**Figure 2:**
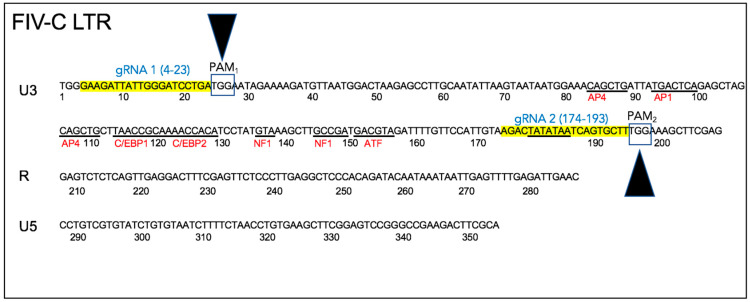
Feline immunodeficiency virus (FIV)-C 5′ and 3′ long terminal repeats (LTR) (U3, R, and U5 regions). The guide RNA sequences are depicted in yellow (gRNA1 and gRNA2) while the two proto-spacer adjacent motif (PAM) sites (PAM_1_ and PAM_2_, endonuclease cut sites) are in boxes and indicated by the black arrowheads. Known transcription factor binding motifs and the TATA box are underlined.

Two conserved Cas9 cleavage sites were identified within the FIV-C U3 region using freely available software (https://www.blueheronbio.com). The two U3 sites were selected based upon (i) proximity to conserved Cas9 PAM cleavage sites (TGG), (ii) relatively strong conservation of the FIV-C viral sequences within the U3 sites through time in a closely monitored population of experimentally FIV-C infected cats [23,24,25], and (iii) the fact that the two chosen cleavage sites essentially encompassed the entire viral U3 promoter region, including the TATA box and all of the known transcription factor binding sites (Figure 2). The FIV sequence-specific gRNAs are depicted in Figure 2—gRNA 1 (5′-GAAGATTATTGGGATCCTGA) and gRNA 2 (5′-AGACTATATAATCAGTGCTT).

**Figure 3:**
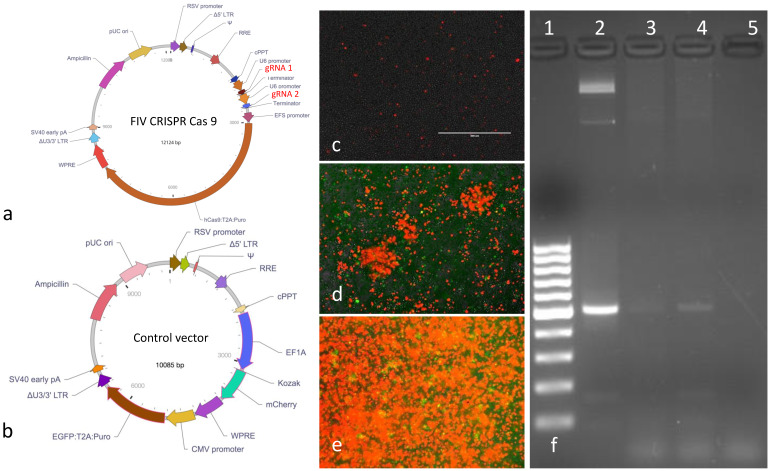
Schematic diagrams of the (**a**) FIV CRISPR Cas9 and (**b**) control vectors. Fluorescent microscopy images of MCH5-4 cells infected with the control vector for 2, 6, or 17 days (**c**, **d**, or **e**, respectively). Images **d** and **e** were obtained during selection (puromycin). (**f**) Ethidium bromide-stained agarose gel electrophoresis of PCR amplicons (FIV CRISPR Cas9 nucleotides 2166-2691). The black arrowhead indicates 500 base pairs (appropriate sized amplicon). Lane 1—molecular weight markers, lane 2—plasmid DNA (positive control), lane 3—FIV CRISPR Cas9 infected/selected feline PBMC, lane 4—feline MCH5-4 cells, lane 5—water template (negative control).

The replication defective, FIV CRISPR Cas9 and control lentiviral vectors were designed around a replication defective lentiviral plasmid backbone (Vector Builder, Figure 3a,b). The vector coding sequences included a puromycin resistance gene (*Puro*) for eukaryotic cell selection and an ampicillin resistance gene (*Ampicillin*) for selection and amplification of the plasmid vectors in *E. coli* bacteria. The gRNAs were expressed under tandem U6 promoters. The control vector encoded the two fluorescent markers mCherry (red fluorescent protein) and enhanced green fluorescent protein (EGFP). The entire nucleotide sequences of both plasmids were confirmed by sequencing (Vector Builder). The two lentiviral vectors were packaged by the vendor and the infectious titers were determined to be 1.87 × 10^9^ or 2.39 × 10^8^ infectious virions/mL for the FIV CRISPR Cas9 or control lentiviral vectors, respectively. Vectors were shipped from the vendor on dry ice in 100 μL aliquots in sterile Hank’s balanced salt solution (HBSS). Vectors were stored at −80 °C until utilized. The FIV CRISPR Cas9 template plasmid was also provided by the vendor in a transformed *E. coli* stock (Stbl3 cells). The FIV CRISPR Cas 9 plasmid was propagated and amplified using a commercial kit following manufacturer’s protocols (Qiagen Plasmid Plus Midi-kit, Redwood city, CA, USA). 

### 2.3. Determination of the Optimal Lentiviral Multiplicity of Infection 

In order to determine the optimal ratio of the CRISPR Cas9 lentivirus to feline cells (multiplicity of infection, or MOI), a series of experiments were performed using varying infectivity ratios of the control lentiviral vector to the cultured feline continuous IL2-independent T-lymphocyte cell line MCH5-4 (gift of Dr. E. Sparger, UC Davis) [26]. Cells were enumerated using a Coulter Analyzer (Beckman Coulter) and were cultured in 24-well tissue culture plates (Costar). The culture media was freshly made: 500 mL RPMI 1640 media (Gibco), 70 mL fetal bovine serum (Gemini Biotec, West Sacramento, CA, USA), 7 × 10^3^ units human recombinant IL2 (NIH AIDS Reagent Program), 700 uL sodium pyruvate (Fisher Scientific, Waltham, MA, USA), 700 uL MEM Vitamin 100× (Corning, Tewksbury, MA, USA). The assessed MOI ratios included 1, 3, 10, or 15 infectious virions per cell. Cells were incubated at 37 °C in 5% CO_2_ incubator. The media was exchanged for fresh media every 48 h for 14 days. From day 4 of incubation on, 0.25 μg puromycin (Sigma, St. Louis, MO, USA) was added per mL of the culture media in order to select for stable integration of the control lentiviral vector into the host cell DNA. Fluorescent images were serially captured using an inverted fluorescent microscope (EVOS FL, Life Technologies, Waltham, MA, USA) and evaluated using Zeiss LSM Image Browser (version 4.2.0.121) software to merge red and green fluorescence images. 

### 2.4. Animals

In order to isolate feline peripheral blood mononuclear cells (PBMC), samples of whole were obtained from specific pathogen free cats from the UC Davis Feline Nutrition Colony. Blood sampling was in accordance with the established IACUC protocol (#19930, approved 10 May 2017, expires 10 May 2020). The study described here adheres to all of the ethical standards put forth by the UC Davis IACUC committee. 

### 2.5. Generation of FIV CRISPR Cas9 Cell Lines 

Feline primary peripheral blood mononuclear (PBMC) cells and feline MCH5-4 cells were infected with the FIV CRISPR Cas9 lentivirus and subsequently treated with puromycin in order to select for stable integration of the CRISPR Cas9 editing tool within these feline cells. Feline primary PBMC were freshly isolated by density centrifugation through Ficoll-Hypaque (Sigma) from whole feline blood obtained via jugular venipuncture from specific pathogen free (SPF) cats (University of California Davis Feline Nutrition Colony). Feline PBMC were cultured in 500 mL RPMI 1640 media (Gibco), 55 mL fetal bovine serum (Gemini Biotec), 5.5 mL Penicillin/Streptomycin 100× solution (Hyclone), 545 uL 2-mercaptoethanol 1000× solution. Then, 1 × 10^4^ units of human recombinant IL2 (NIH AIDS Reagent Program) were added per 100 mL of the media.

Approximately 9 × 10^6^ MCH5-4 cells or feline PBMC were infected with 1.8 × 10^8^ FIV CRISPR Cas9 lentivirus in 4 mL cultures (MOI 20). The cells and lentivirus were gently swirled for 30 s every 15 min for 1 h. The media was exchanged for fresh media on day 2. On day 4, the infected cells were treated with 0.25 μg puromycin per mL media (selective media). The media was exchanged for fresh selective media every 2–3 days for a total of 17 days (end of selection). At the end of selection, genomic DNA (gDNA) was isolated from a subset of cells from each cell line (1 × 10^6^ cells, QIAamp DNA Minikit, Qiagen) and the balance of the selected cells were cryopreserved in freezing media (10% DMSO, 90% FBS) and archived in liquid nitrogen for subsequent use (Experiments 2 and 3). Standard PCR was performed with the selected-cell DNA in order to identify the presence of the FIV CRISPR Cas9 coding sequence using primers spanning the CRISPR Cas9 gRNA region (nucleotides 2166-2691). The sequences of the forward and reverse primers were—CRISPR For 5′-CGAAACACCGGAAGATTATTGGGATCC and CRISPR Rev 5′CGCGCTAAAAACGGACTAGC. Standard PCR was performed in a thermocycler (Mastercycler gradient, Eppendorf) using Taq DNA polymerase (Invitrogen) and the following cycling conditions- 95 °C for 2 min, followed by 30 cycles of 95 °C for 15s, 54 °C for 1 min, 68 °C for 1 min and a final extension step of 68 °C for 5 min. Water template served as a negative control while plasmid DNA served as the positive control. The resulting PCR products were electrophoresed onto ethidium bromide-stained agarose gel. All subsequent experiments were performed with MCH5-4 cells. 

### 2.6. Real-Time PCR Assays

DNA was isolated from cultured feline MCH5-4 cells and RNA was isolated from cell free supernatant using commercial kits (see Experiments 1–3, below). The isolated RNA was DNAse treated (TURBO DNase, ThermoFisher) and reverse transcribed to cDNA (First Strand cDNA Synthesis kit, Origene). Cell-derived FIV *gag* DNA and cell free cDNA were quantified using real-time PCR. Assays to detect and quantify FIV *gag* and feline GAPDH (in order to normalize the amount of the viral Gag) were performed using either an Applied Biosystems 7300 Real-Time PCR System with SYBR green PCR Master Mix (Applied Biosystems) and 25 μL reactions (Experiments 1 and 2) or an Applied Biosystems QuantStudio 3 Real-Time instrument and PowerUp SYBR Green Master Mix (Thermo Fisher Scientific) following the manufacturer’s recommended protocols for 10 μL reaction (Experiment 3). All FIV *gag* DNA or cDNA templates were amplified using the previously published primers FIV QT gag for (5′-TAGCCCTTGACCCAAAAATG), FIV QT gag rev (5′-ATTGGCCGAAAAAGCTGTAA) yielding a 120 bp amplicon [24]. Feline GAPDH was quantified in parallel samples in order to normalize the results of FIV gag proviral DNA and cell-associated RNA. Feline GAPDH primers included the forward primer 5 GAPDH (5′-AAATTCCACGGCACAGTCAAG) and the reverse primer 3 GAPDH (5′-TGATGGGCTTTCCATTGATGA) [24].

For amplifying FIV gag, the following cycling conditions were utilized for 7300 Real-Time PCR system (Applied Biosystem)—50 °C for 2 min, 95 °C for 10 min, followed by 40 cycles of 95 °C for 15 s, 58 °C for 30 s, 68 °C for 30 s. The cycling conditions for amplifying GAPDH on the 7300 system were 50 °C for 2 min, 95 °C for 10 min, followed by 40 cycles of 95°C for 15 s, 58 °C for 30 s, 95 °C for 15 s, 60 °C for 1 min, 95 °C for 15 s, and 60 °C for 15 s. The following cycling conditions were run with the QuantStudio 3 (Applied Biosystem). For amplifying FIV gag—50 °C for 2 min, 95 °C for 2 min, followed by 40 cycles of 95 °C for 15 s, 60 °C for 1 min. The cycling conditions for amplifying GAPDH using the QuantStudio 3 were as follows—50 °C for 2 min, 95 °C for 2 min, followed by 40 cycles of 95 °C for 15 s, 58 °C for 30 s, 72 °C for 1 min.

For all of the real time PCR assays, unknown samples were run in parallel triplicate repeats, FIV gag plasmid DNA was run in parallel as a positive control and water template was run as a negative control. For cDNA samples, a sample lacking reverse transcriptase (RT-) was included as an additional negative control. In all cases, the amplification cycles were followed by a melting curve to control for primer specificity. FIV gag and feline GAPDH copy number was calculated using a linear regression equation generated by our laboratory. The FIV gag copy number was then normalized to GAPDH and expressed as copies of FIV gag per 10^6^ copies of GAPDH [24]. Cell-free supernatant associated viral RNA (cDNA) was normalized to the initial volume of the culture fluid in mL.

### 2.7. Experiment 1—Assessing whether or not FIV-Infected Cells Subsequently Treated with FIV CRISPR Cas9 Lentiviral Vector Results in a Reduction of FIV Provirus and Cell Free Virus

**Figure 4:**
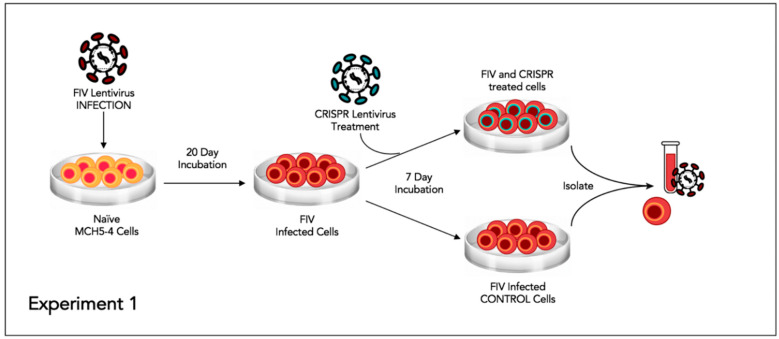
Schematic diagram of Experiment 1.

This experiment was designed to assess the effect of the CRISPR Cas9 gene editing tool on attenuating the FIV proviral load and cell free virus production in a population of chronically FIV-infected cells (Figure 4). 

Approximately 6 × 10^6^ feline MCH5-4 cells were infected with 1 × 10^8^ copies (MOI 16, measured using real-time PCR) of in vitro passaged FIV-C [24] in a vertically oriented T25 tissue culture flask (Corning). The cells and virus were gently swirled for 30 s every 15 min for 1 h. The infected MCH5-4 cells were incubated for 20 days with a complete media exchange on days 2, 5, 10, 15, and 20. On day 20, the chronically FIV-C infected MCH5-4 cells were divided into 6 individual wells, with 1 × 10^6^ cells per well in a 48-well tissue culture plate (Corning). Three of the wells were subsequently infected with FIV CRISPR Cas9 lentivirus (Treated cells) at an MOI of 20 (2 × 10^7^ infectious virions/well) while 3 wells were not infected with the FIV CRISPR Cas9 lentivirus (Control cells). The FIV CRISPR Cas9 lentivirus was added dropwise to the three treated wells and the plate was gently swirled every 15 min for one hour. The media was exchanged with fresh media on days 2 and 5. On Day 7, the cells were individually collected from each well for DNA isolation (AllPrep DNA/RNA Mini Kit, Qiagen) and the centrifuged supernatant was collected for cell-free viral RNA isolation (QIAamp viral RNA Mini Kit, Qiagen). Isolated nucleic acids were prepared for real time PCR (cells, DNA) or real-time RT PCR (supernatant, RNA), as described above. 

### 2.8. Experiment 2—Effect of FIV CRISPR Cas9 Stably Infected Cells Subsequently Infected with FIV 

**Figure 5:**
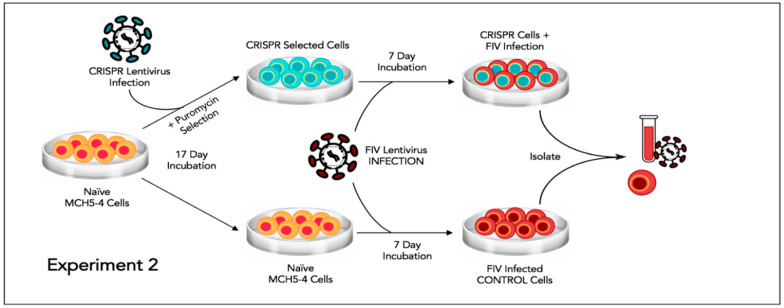
Schematic diagram of Experiment 2.

In this experiment, a population of MCH5-4 cells that had been selected for stable integration of the RNA-directed gene editing tool was subsequently infected with FIV-C and assessed for the effect on the FIV proviral load and cell free virus production (Figure 5). 

Two million naïve MCH5-4 cells or FIV CRISPR Cas9 lentivirus infected and selected MCH5-4 cells (see generation of FIV CRISPR Cas9 cell lines, above) were infected with 7 × 10^7^ FIV-C virions (MOI 35, measured using real-time PCR) in a 12 well tissue culture plate (Corning, Tewksbury, MA, USA). In previous experiments, we have determined that an MOI of 15–35 FIV-C virions results in approximately 100% infection of the cultured MCH5-4 cells. Each experimental treatment was performed in parallel and in triplicate. Then, 200 μL of fresh culture media without puromycin was added to each well every 2 days. On Day 7, the cells were individually collected from each well for isolation of DNA (AllPrep DNA/RNA Mini Kit, Qiagen) while the cell-free supernatant was collected for isolation of viral RNA (QIAamp viral RNA Mini Kit, Qiagen). Isolated nucleic acids were prepared for real-time PCR (cells, DNA) or real-time RT PCR (supernatant, RNA), as described previously. 

### 2.9. Experiment 3—Effect of Gene Editing on the Production of Infectious Virions 

**Figure 6:**
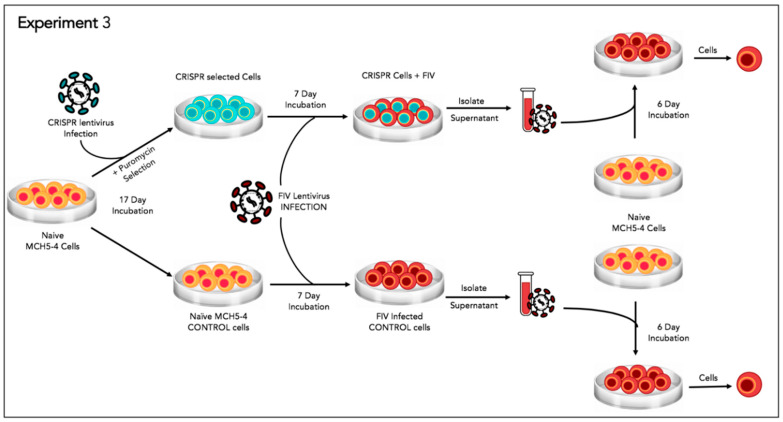
Schematic diagram of Experiment 3.

In order to determine the effect of the FIV-targeted gene editing tool on the production of infectious virions, a two-step infection experiment was performed (Figure 6). In the first infection, approximately two million naive MCH5-4 cells or FIV CRISPR Cas9 lentivirus infected and selected MCH5-4 cells (see generation of FIV CRISPR Cas9 cell lines, above) were infected with 4.2 × 10^6^ copies (measured using real-time PCR) FIV-C per well. Each experimental treatment was performed in parallel and in triplicate. On days 1, 3, 4, and 5 following FIV infection, the culture media was replaced with fresh culture media. In order to assess the infectivity of the produced virions, a second infection step was performed on day 6. One milliliter of cell-free supernatant harvested individually from each well (presumably containing infectious virus) was passaged onto naïve MCH5-4 cells (1.3 × 10^6^ cells/well) in a 12 well tissue culture plate (Costar). These cultures were maintained for 6 additional days with complete media exchanges on days 2 and 4. On day 6, the cell-associated DNA was isolated using a commercial kit (Quick DNA/RNA Miniprep Kit, Qiagen) following the option for DNA isolation only. Proviral gag and GAPDH were quantified using real time PCR as described previously. 

### 2.10. Data Analysis

The mean and standard deviation of three identical but separate experiments were graphed as a bar (mean) with error bars (standard deviation). T tests were not performed due to the small number of experimental replicates. 

## 3. Results

### 3.1. Determination of the Optimal Multiplicity of Infection and Cell Line

Fluorescence microscopy images of MCH5-4 cells infected with the control lentivirus vector at an initial MOI of 15, imaged at day 2 (prior to puromycin selection) and days 6 and 17 (after puromycin selection) are shown in Figure 3c–e, respectively. In these images, a progressively increased percentage of cells demonstrate bright red cytoplasmic fluorescence, indicating ample expression of the mCherry transgene. At the end of selection (day 17), 90–100% of the cells in the image demonstrate red fluorescence. These results are consistent with a progressively increasing proportion of cells containing the control lentiviral vector as a result of puromycin selection (i.e., successful selection). Interestingly, relatively few cells demonstrate green fluorescence (EGFP transgene expression) in this panel of images. We suspect that this is the result of mCherry having its own EF1-alpha promoter (EF1A) while the EGFP gene is linked to the puromycin gene (Puro) via a self-cleaving peptide (T2A, Figure 3b). Prior studies have indicated that protein expression decreases by approximately 70% at the second gene position compared to the first gene position in genes linked by a self-cleaving peptides [27].

Experiments using the control lentivirus at an MOI of 1, 3, 10, or 15 resulted in a dose-dependent percentage of cells expressing the mCherry and EGFP transgenes: MOI 1—least expression, MOI 15—most expression. Although the available amount of control lentivirus precluded testing at a virus-to-cell ratio greater than MOI 15, we chose to use an MOI of 20 as the dose for the FIV CRISPR Cas9 lentivirus infections for unselected cells in Experiment 1 and puromycin selected cells in Experiments 2 and 3. This choice was based upon (i) the availability of a greater amount of FIV CRISPR Cas9 lentivirus and (ii) the dose-dependent trend of transgene expression using the control lentivirus suggested that MOI 20 would be optimal. 

After 17 days of selection, DNA was isolated from CRISPR Cas9 lentivirus infected and puromycin selected feline PBMC and MCH5-4 cells. Standard PCR using the CRISPR forward and reverse primers was performed in order to detect the presence of the cellular CRISPR Cas9 DNA. Resulting PCR amplicons were electrophoresed on an ethidium bromide-stained agarose gel (Figure 3f). Appropriately sized amplicons (arrowhead) are evident in lanes 2 (plasmid positive control), 3 (feline PBMC DNA), and 4 (MCH5-4 DNA) but not in lane 5 (water template, negative control). In Figure 3f, The MCH5-4 derived amplicon is markedly brighter than the feline PBMC derived amplicon, consistent with successful selection and stable integration of the CRISPR Cas9 tool within MCH5-4 cells. As a result of this finding, the MCH5-4 cell line was used in all of the subsequent experiments. 

### 3.2. Experiments 1, 2, and 3

**Figure 7:**
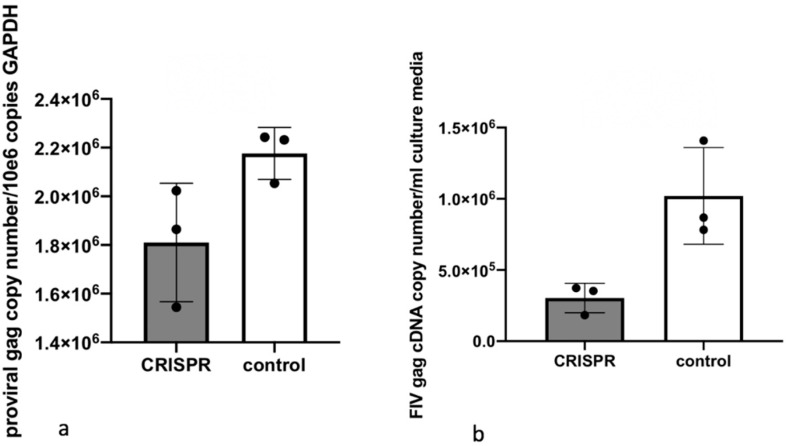
Experiment 1. (**a**) In chronically FIV-infected MCH5-4 cells (infected for 20 days with FIV followed by 7 days of infection with FIV CRISPR Cas9), cells treated with the CRISPR gene editing tool (grey bars) have a reduction in the mean proviral DNA relative to untreated control cells (white bars). (**b**) Cell-free FIV is reduced in culture media derived from cells treated with the CRISPR reagent relative to untreated control cells. The bar represents the mean while the error bars represent the standard deviation of three parallel experiments.

In Experiment 1, chronically FIV-C infected MCH5-4 cells subsequently infected with the FIV-specific CRISPR Cas9 lentivirus demonstrated a slight 1.3-fold reduction in the mean cell associated FIV proviral DNA in CRISPR Cas9 lentivirus treated versus untreated cells (Figure 7a). A 3.4-fold reduction of supernatant-derived cell free virus (CRISPR, Figure 7b) was also identified relative to chronically FIV-C infected cells that were not treated with the CRISPR Cas9 lentivirus (control). 

**Figure 8:**
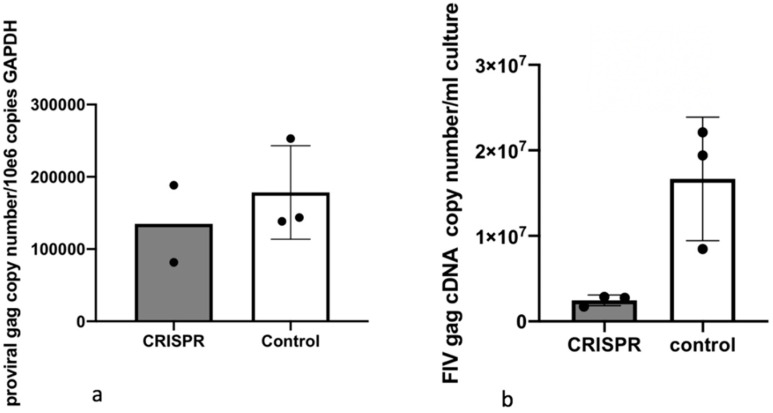
Experiment 2. (**a**) MCH5-4 cells selected for stable integration of the FIV CRISPR Cas9 editing tool and subsequently infected with FIVC (grey bar). One of the CRISPR-treated DNA samples was inadvertently lost during processing (grey bar, two data points). (**b**) Cell-free FIV is reduced in culture media derived from the cells treated with the CRISPR reagent relative to untreated control cells (6.8-fold reduction). The bar represents the mean while the error bars represent the standard deviation of three parallel experiments.

In Experiment 2, the mean proviral DNA copy number was reduced for CRISPR Cas9-treated cells relative to control cells (however, only two experimental units were available for analysis) (Figure 8a). MCH5-4 cells that were previously selected for integration of the CRISPR Cas9 construct followed by infection with FIV-C had a 6.8-fold reduction in the amount of FIV-C cell free viral RNA (CRISPR, Figure 8b) relative to MCH5-4 cells that lacked the CRISPR Cas9 construct (control). 

In Experiment 2, the average viral gag copy number were determined to be approximately 10-fold greater in both CRISPR treated and control wells relative to comparable wells in Experiment 1. This is likely due to the difference between an acute FIV infection (Experiment 2) and chronic infection (Experiment 1). 

**Figure 9:**
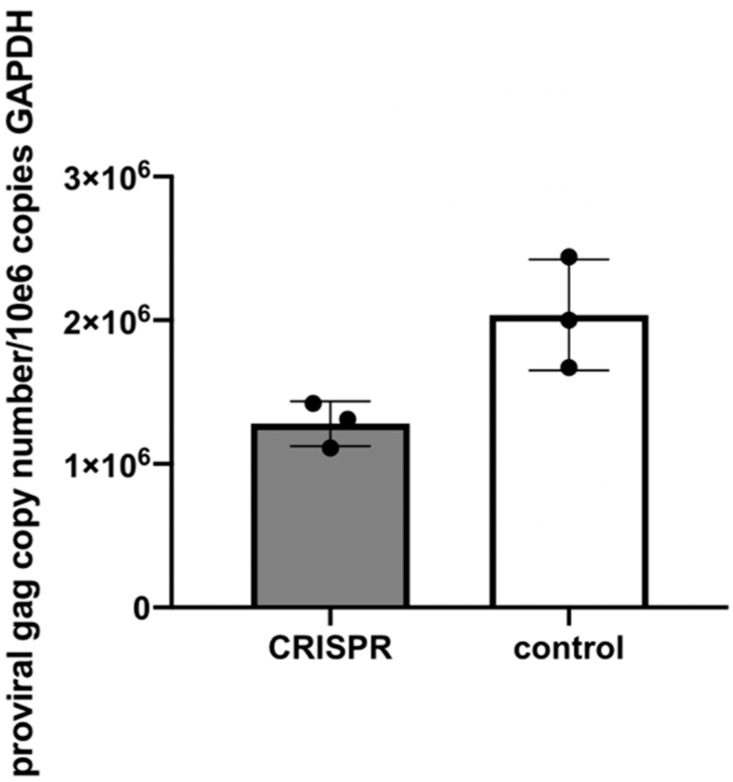
Experiment 3. In a two-step infectivity experiment, the proviral DNA of MCH5-4 cells infected with cell free FIV harvested from a stably transduced CRISPR Cas9 cell line (grey bar) have a 1.6-fold reduction in proviral DNA relative to control cells (white bar). The bar represents the mean while the error bars represent the standard deviation of three parallel experiments.

In Experiment 3, the two-step infectivity experiment, cell free FIV harvested from MCH5-4 cells infected and selected for integration of the FIV CRISPR Cas9 gene editing tool had attenuated infectivity as determined by a reduced proviral load in cells infected with virus derived from CRISPR-treated cells relative to control cells (1.6-fold reduction, Figure 9). Only proviral DNA was assessed in this assay in order to provide a measure of true infection. That is, viral RNA, if measured, might represent inoculating, non-infectious virus.

## 4. Discussion

In this set of experiments, we have demonstrated that a CRISPR Cas9 gene editing tool targeting the FIV-C U3 region reduced cell-free FIV RNA and the infectious potential of virions produced by cells treated with the gene editing tool relative to untreated control cells. However, the reduction of FIV proviral DNA in the CRISPR treated cells was minimal relative to control cells. This may be the result of suboptimal experimental timing (e.g., excessively prolonged/brief incubation periods) or suboptimal FIV dose. Another possibility is that the putatively excised FIV *gag-pol-env* coding region may actually continue to exist within the cells at the point of nucleic acid isolation as an excised linear fragment or circularized segment of proviral DNA (episome). As a result, experimental isolation of the cell-associated DNA followed by quantification of proviral *gag* using real-time PCR might fail to identify a significant difference relative to untreated control cells (however, the DNA isolation kit indicates an optimal DNA fragment size recovery of 15–30 kb; the excised proviral DNA is ~8 kb). A third possibility is that the CRISPR strategy employed here may have resulted in a partial (~171 nucleotide) deletion of U3 promoter between the two PAM sites (PAM_1_ at LTR position 27 and PAM_2_ at position 198), leaving the rest of the integrated proviral sequence (including *gag*) intact (Figure 2). In such a scenario, the CRISPR Cas9 induced U3 deletion would be expected to compromise viral transcription (and as a result, virion production) due to a loss of the U3 transcription factor binding sites and TATA box, leading to the spectrum of results we identified here. 

The reduction of proviral DNA for the two-step FIV infection study in Experiment 3 was modest (1.6-fold reduction in proviral DNA with CRISPR Cas9 treatment). This again may be the result of suboptimal experimental timing (length of incubation periods). It is also possible that the acute FIV infection strategy we employed may have simply set the bar “too high” for detecting meaningful viral attenuation/eradication. That is, the quantity (dose) of FIV-C used in these experiments (MOI of 35–50) may have overwhelmed the functionality of the gene editing method to markedly attenuate the provirus. It is very possible that this lentiviral gene editing strategy might prove to be more effective in latently FIV-infected cells. In chronically infected animals, the persistently and latently infected cells are the target for therapeutic genetic editing. However, latently infected cells are rare in chronically immunodeficiency virus-infected animals. In a colony of experimentally FIV-infected cats, we determined that the latent reservoir in peripheral CD4+T cells during the asymptomatic phase of FIV-C infection to be approximately one in 10^5^ cells (that is, 1 in 10^3^ cells is infected, but only 1 in 10^2^ of those is replication competent) [28]. In HIV infected human patients on antiretroviral therapy, the number of latently infected peripheral CD4+ T cells ranges from 1 to 10 cells per 10^6^ [28]. Viremia in well controlled HIV-infected patients can be close to undetectable. Clearly, the viral replication dynamics in chronically infected hosts differs markedly from the in vitro infectious assays modeled here. 

We hypothesized that the gene editing strategy would excise the entire FIV coding region (*gag-env*), leaving a remnant viral LTR integrated into the feline genomic DNA. It is possible that this remnant viral LTR (promoter) might promote aberrant expression of neighboring host genes, possibly resulting in unregulated cell growth. For this reason, we employed a combination of two different gRNAs targeting the 5′ and 3′ ends of the FIV U3 region. Deletion of this region should quench the promoter functionality and prevent activation of adjacent host coding regions. Another concern is the potential persistence of the excised viral coding region as a circularized episome within the cell. Could the excised viral coding region be subsequently reintroduced into the genome via some form of genetic recombination? Ultimately, these issues will need to be resolved in an animal model of immunodeficiency virus infection, such as the FIV-infected cat. 

Limitations of this work include a lack of whole genome sequencing data to identify (or fail to identify) (i) evidence of CRISPR-associated genotoxicity, (ii) direct evidence of FIV proviral genome excision in CRISPR treated cells. Although we hypothesized that this gene editing strategy would result in excision of the FIV provirus from the host cell genome, we were not able to provide direct evidence for this actually occurring. Multiple unsuccessful attempts were made using standard PCR/cloning and inverse PCR in order to identify (i) the presence or absence of genetic modification of the integrated provirus, and (ii) determine if excised, circularized episomal proviral DNA was present in CRISPR treated cells. Whole genome sequencing would potentially solve these detection issues. Another limitation of this work was that control cells in Experiments 1–3 were not infected with a control lentivirus. Unfortunately, the quantity of control lentivirus available for experimental use was limited. In addition, a limited supply of the FIV CRISPR Cas9 lentivirus limited the number of experimental replicates that could be performed and as a result of the small number of experimental replicates, data are portrayed as means and standard deviations without testing for significance. 

Multiple barriers need to be surmounted in order to meaningfully reduce or remove the integrated proviral genomes from infected cells (proviral attenuation). Proviral attenuation in an infected host requires maximizing the efficiency of delivery of the CRISPR Cas9 nuclease system to the infected cells, along with concurrent prevention of off-target effects within the host cell genome (genotoxicity). The precise method of introducing the editing nucleases into these rare, latently infected cells is the key barrier. This process requires the creation of a gene delivery system that efficiently delivers the CRISPR Cas9 editing machinery to any and all host cells, or alternatively, specifically targets latently infected host cells. The difficulty with the latter strategy is that, by definition, lentivirus-infected cells in latency do not express viral antigens, making their detection challenging. Although at present, both of these approaches appear to be problematic, a partial reduction of the proviral load in key reservoir cells may have clinical benefits. 

## 5. Conclusions

We have shown that a CRISPR Cas9 gene editing tool targeting the FIV-C U3 region significantly reduced cell-free FIV RNA and the infectious potential of virions produced by cells treated with the gene editing tool relative to untreated control cells. This study represents the initial steps towards the development of an effective method of proviral eradication in an immunodeficiency virus-infected host. For this gene editing strategy to be successful, a number of hurdles must be surmounted: (i) in vitro incubation times/viral challenge dose needs to be further optimized, (ii) ex vivo experiments using animal-derived latently infected cells need to be performed that demonstrate an effective attenuation/elimination of proviral loads, (iii) a highly efficient method of infecting (or transducing) these rare lentivirus infected cells in vivo needs to be optimized, and (iv) the potential of therapy-associated off-target genotoxicity needs to be minimized or eliminated. We contend that the FIV-infected cat is an ideal model for the testing of these experimental manipulations.
